# Respiratory consequences of N95-type Mask usage in pregnant healthcare workers—a controlled clinical study

**DOI:** 10.1186/s13756-015-0086-z

**Published:** 2015-11-16

**Authors:** Pearl Shuang Ye Tong, Anita Sugam Kale, Kailyn Ng, Amelia Peiwen Loke, Mahesh Arjandas Choolani, Chin Leong Lim, Yiong Huak Chan, Yap Seng Chong, Paul Anantharajah Tambyah, Eu-Leong Yong

**Affiliations:** Department of Obstetrics and Gynecology, National University Hospital, 11 Mandalay Road, Singapore, 308232 Singapore; Lee Kong Chian School of Medicine, Nanyang Technological University, Singapore, 11 Mandalay Road, Singapore, 308232 Singapore; Biostatistics Unit, National University of Singapore, Singapore, Republic of Singapore; Medicine, National University of Singapore, 1E Kent Ridge Road, Level 12, Singapore, 119228 Singapore

**Keywords:** N95 respirators, Infection control, Pregnant women, Healthcare workers, Respiratory parameters, Controlled trial

## Abstract

**Background:**

Outbreaks of emerging infectious diseases have led to guidelines recommending the routine use of N95 respirators for healthcare workers, many of whom are women of childbearing age. The respiratory effects of prolonged respirator use on pregnant women are unclear although there has been no definite evidence of harm from past use.

**Methods:**

We conducted a two-phase controlled clinical study on healthy pregnant women between 27 to 32 weeks gestation. In phase I, energy expenditure corresponding to the workload of routine nursing tasks was determined. In phase II, pulmonary function of 20 subjects was measured whilst at rest and exercising to the predetermined workload while breathing ambient air first, then breathing through N95-mask materials.

**Results:**

Exercising at 3 MET while breathing through N95-mask materials reduced mean tidal volume (TV) by 23.0 % (95 % CI −33.5 % to −10.5 %, *p* < 0.001) and lowered minute ventilation (VE) by 25.8 % (95 % CI −34.2 % to −15.8 %, *p* < 0.001), with no significant change in breathing frequency compared to breathing ambient air. Volumes of oxygen consumption (VO_2_) and carbon dioxide expired (VCO_2_) were also significantly reduced; VO_2_ by 13.8 % (95 % CI −24.2 % to −3 %, *p* = 0.013) and VCO_2_ by 17.7 %, (95 % CI −28.1 % to −8.6 %, *p* = 0.001). Although no changes in the inspired oxygen and carbon dioxide concentrations were demonstrated, breathing through N95-mask materials during low intensity work (3 MET) reduced expired oxygen concentration by 3.2 % (95 % CI: −4.1 % to −2.2 %, *p* < 0.001), and increased expired carbon dioxide by 8.9 % (95 % CI: 6.9 % to 13.1 %; p <0.001) suggesting an increase in metabolism. There were however no changes in the maternal and fetal heart rates, finger-tip capillary lactate levels and oxygen saturation and rating of perceived exertion at the work intensity investigated.

**Conclusions:**

Breathing through N95 mask materials have been shown to impede gaseous exchange and impose an additional workload on the metabolic system of pregnant healthcare workers, and this needs to be taken into consideration in guidelines for respirator use. The benefits of using N95 mask to prevent serious emerging infectious diseases should be weighed against potential respiratory consequences associated with extended N95 respirator usage.

**Trial Registration:**

The study was registered at clinicaltrials.gov, identifier NCT00265926.

## Background

Lessons learnt on infection control from the Severe Acute Respiratory Syndrome (SARS) pandemic in 2003 have been used to formulate strategies [1, 2] to manage the recent Middle Eastern respiratory syndrome (MERS) [3] and H7N9 influenza outbreaks [4]. These infection control measures include recommendations for increased use of protective filtering face-piece respirators (FFR), such as N95-masks [5] especially during aerosol generating procedures. Existing influenza pandemic control plans in many countries have also incorporated recommendations for more widespread use of FFR [6–8]. When the influenza A H1N1 pandemic was declared in 2009, there were guidelines for universal use of N95-masks despite a lack of scientific evidence for its appropriateness in different health care settings [9, 10]. The N95 FFR has also been recommended for the novel MERS Coronavirus.

Little is known about the effects of N95-masks on the respiratory function of pregnant healthcare workers, who can be subjected to prolonged usage of FFR because of their vulnerability to complications from influenza, varicella, and other pathogens transmitted via the respiratory tract [11]. It is also known that pregnant women have a significantly greater respiratory burden due to factors such as increased oxygen (O_2_) demand, increased nasal airway resistance, decreased functional residual capacity due to diaphragmatic splinting; all these contributing to the “physiologic” dyspnea of pregnancy [12]. There are also robust data linking respiratory compromise and adverse perinatal outcomes in women who have chronic respiratory conditions, from large scale studies on women with conditions such as asthma and obstructive sleep apnea. These outcomes include preterm labour, impaired fetal growth, and pre-eclampsia [13, 14].

Balancing the potential benefits of respiratory protection against the possible discomfort [15] and potential additive adverse effects on the respiratory functions of pregnant healthcare workers is difficult in the absence of clear data although there is no definite evidence of harm from decades of use of such respirators [16]. A recent study comparing a cohort of pregnant women between 13 to 35 weeks gestation and non-pregnant women showed no differences in respiratory rate, oxygen saturation and transcutaneous carbon dioxide levels in pregnant compared with non-pregnant subjects wearing the N95 FFR during exercise and sedentary activities for over a 1-hour period [17]. However, that study did not specifically examine the impact on busy healthcare workers. Pregnancy has been reported to be the most common cause for denying medical clearance for N95-mask use in a non-medical setting but the specific adverse effects of the respirator itself have not been documented [18]. Our study was performed to address the limited data on N95-mask usage in pregnancy with the aim of investigating the effects of breathing through the N95 mask materials on respiratory functions at rest, during low intensity work, and recovery thereafter in pregnant healthcare workers. The differences in work of breathing and potential adjustments in respiration that are contributed by pregnancy may provide guidance on the use of N95-masks by pregnant health care workers in high-risk environments.

## Methods

This controlled clinical trial was carried out in 2 phases in the Investigational Medicine Unit of National University Hospital (NUH), Singapore. Study procedures were approved by the National Healthcare Group Domain Specific Review Board in July 2010 (Reference Number: 2010/00226), and written informed consent was obtained from all participants.

Healthy women with singleton pregnancies between 27 to 32 weeks gestation were recruited on a voluntary basis from amongst hospital staff and clinic patients. Eight pregnant health care workers were recruited in phases I and 20 pregnant women were recruited in phase II to participate in the study. Subjects were instructed to have adequate rest and to avoid strenuous activity prior to the study to ensure that the tests were conducted under normal lifestyle conditions. All subjects were told to have their meals at least 2 h before start of study. A screening questionnaire was administered to each subject, who then had baseline medical and obstetric examinations prior to participating in the study.

### Inclusion and exclusion criteria

Subjects had spontaneously conceived singleton pregnancies and were between 21 to 40 years old. They had no history of cardiorespiratory illness, influenza-like illness in the week prior to the trial, or any pregnancy-related complications such as gestational diabetes, hypertension, intrauterine growth restriction, placenta praevia, ruptured membranes, or threatened preterm labor. They were also free of any neuromuscular conditions that would preclude them from using the treadmill. Their hemoglobin levels were ≥11g/dL, and they did not have any haemoglobinopathies such as thalassemia that could interfere with oxygen carriage in the blood.

### Study design

**Fig. 1:**
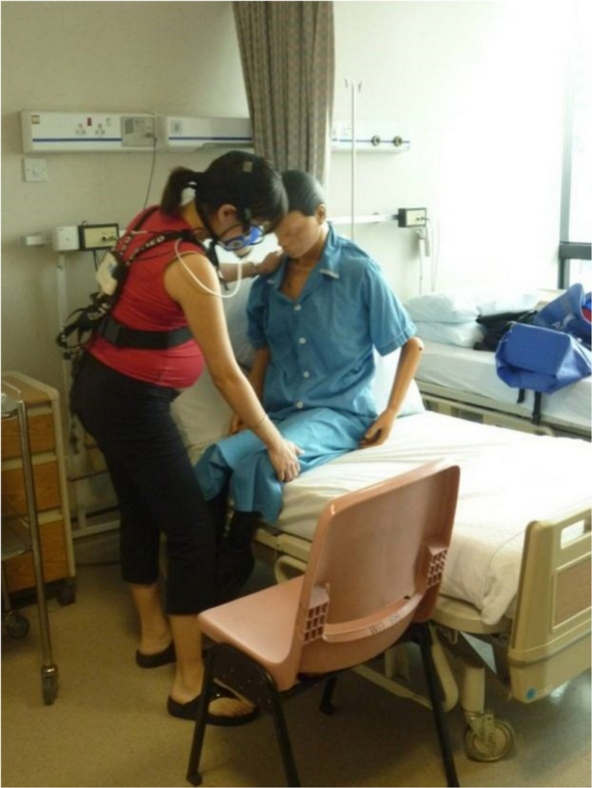
Determination of average work intensity of Health care workers: In phase I pregnant subjects performed simulated patient care activities while breathing through a tight fitting mask with a pneumotachometer. Oxygen content was sampled at every breath and measured with a portable telemetric metabolic cart

In Phase I, the volume of O_2_ uptake (VO_2_) corresponding to the workload of routine nursing tasks in the ward was determined. These healthcare workers wore, and breathed through, a tight-fitted respiratory mask (Hans Rudolph, V-mask, Kansas) that was attached by a harness to a portable telemetric metabolic cart [19–21] (K4b2, Cosmed s.r.l, Rome, Italy) while moving about freely performing simulated routine nursing tasks in a specific order, such as walking around the ward, sponging and transferring mannequins from beds to chairs with another assistant (Fig. 1). Their average work intensity was determined with VO_2_ (ml/kg/min) measurements and converted to the corresponding metabolic equivalents (MET) to gauge the energy expenditure (1 MET is equal to 3.5 ml/kg/min of O_2_ consumed).

**Fig. 2:**
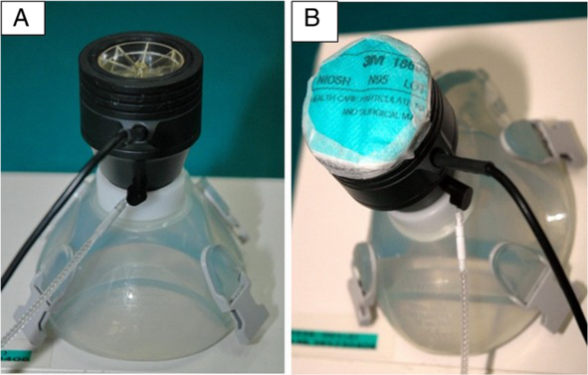
Tight fitting Hans Rudolph respirator masks used in Phase II. (**a**) Control cycles with outlet open to air, and (**b**) N95 cycles with outlet covered by N95 mask materials

In Phase II, the respiratory effects of wearing N95 masks were examined. Each subject underwent two 15-minute exercise cycles on a treadmill. Each subject wore a Hans Rudolph mask, similar to that in Phase I, attached to a laboratory-based metabolic cart (Cortex Metalyser 3BR2, Leipzig, Germany) in order to obtain real time respiratory parameters during exercise. In the first (control) cycle, subjects wore the Hans Rudolph mask with the outlet opened to ambient air. In the second (N95) cycle, outlets of the Hans Rudolph masks were covered by materials obtained from representative supplies of N95 masks (3M, St. Paul, MN, USA). N95-mask materials were trimmed to form an airtight seal over the Hans Rudolph mask outlet so that the air flow resistance on inspiration and expiration would come from the mask material, simulating the actual wearing of an N95 respirator (Fig. 2) This experimental design allowed each subject to act as her own control.

**Fig. 3:**
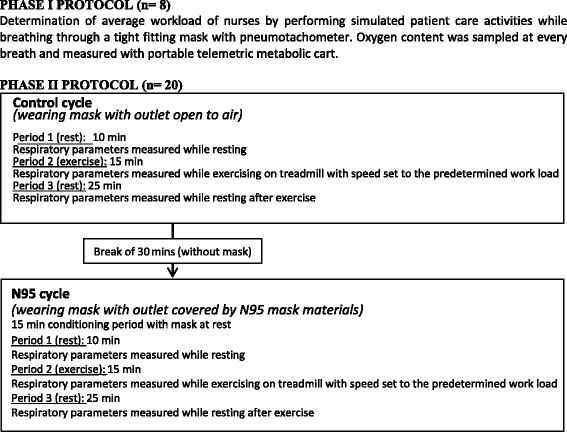
Phase I & 2 Protocols

The fine adjustments were made to the treadmill speed every 3 min to maintain energy expenditure at 3 MET. Similar treadmill speed profile was repeated in the second exercise cycle for each subject. For both Control and N95 cycles, respiratory parameters were measured during an initial 10-minute rest period, followed by a 15-minute exercise period, and subsequently a 25-minute rest period. There was a 30-minute break between control and N95 cycles. Prior to the N95 cycle, an additional 15-minute conditioning period was allowed to enable patient to adapt to N95-respirator conditions. The subjects breathed through the N95 mask materials continuously throughout the N95 cycles (Fig. 3).

A cardiotocography (CTG) was performed prior to the study and after each exercise-cycle. Finger-prick lactate concentrations were measured immediately before and after each exercise-cycle with Lactate Pro (Arkray Global Business Inc). The Borg Scale questionnaire [22] was administered after each exercise cycle to measure the rating of perceived exertion. The Borg Scale ranges from 6 for "no feeling of exertion," to 20 which corresponds to "very, very hard exertion". A fully equipped resuscitation cart was present throughout the study with trained medical personnel available to immediately address any medical concerns of the subjects.

### Equipment for measurement of pulmonary function and its calibration

In both phases, the participants wore a tight fitting mask (Hans Rudolph) that was attached to the metabolic cart through an air sampling tube. Inspired ambient air and expired air were channeled through a pneumotachometer that was attached to the front of the mask which calculated air volume by the rate of rotation of a rotor turbine located within it. The turbine had zero resistance to air flow and the rate of rotation of the turbine, sensed by infrared light within the pneumotachometer, corresponds directly to inspired and expired air volume for each breath. Multiple air samples from each expired-breath was drawn into the metabolic carts through a sampling line for the measurement of oxygen and carbon dioxide content by the respective gas sensors within the metabolic carts. From this data, the following parameters were calculated: volumes of oxygen (VO_2_) and carbon dioxide (VCO_2_) exchanged, breathing frequency (BF), tidal volume (TV), minute ventilation (VE), forced expired O_2_ (FeO_2_), forced expired CO_2_ (FeCO_2_), forced inspired end-tidal O_2_ and CO_2_ concentrations (Fi_et_O_2_, Fi_et_CO_2_).

The calibration procedures for both portable and laboratory-based metabolic carts were carried out according to the manufacturer’s instructions and were performed daily to ensure uniformity in their measurements. The study was conducted in a standardized air-conditioned room similar to a hospital ward with constant humidity and temperature.

### Criteria for study termination

The study was to be terminated if: (1) the CTG revealed that the fetus was adversely affected by the mother's activity on the treadmill either by a suspicious or pathological trace as defined by the National Institute for Health and Care Excellence [23],(2) the subject was unable to complete the trial for any reason including breathlessness or pain, (3) injury was sustained as a result of the exercise, or (4) maternal heart rate was >155 beats/min [24, 25].

### Statistical analysis

Since there were many respiratory variables of interest, the sample size calculations were performed on an overall picture that there was at least a 20 % difference between breathing through N95 mask materials vs control for any of respiratory variable of interest. Postulating that breathing through N95 mask materials would have an at least 20 % variation (with standard deviation 25 %) from the control respiratory variables, recruitment of 20 subjects would have a 90 % power and a 2-sided p-value of 5 % to show a statistically significant result. A mixed linear model analysis (to handle paired observations) adjusting for relevant covariates was performed. All analyses were performed using IBM SPSS version 20.0 (Armonk, NY) and statistical significance was set at *p* < 0.05.

## Results

Twenty-eight pregnant women were recruited between September 2010 and September 2011. In Phase I, the mean and SEM of VO_2_ was 9.04 (±0.75) ml/kg/min, which was equivalent to about ~3 MET. All 8 subjects enrolled in Phase I fulfilled the inclusion criteria and completed the study. Subjects in Phase II were then subjected to this workload. For Phase II, 23 subjects were screened. Two subjects were excluded because of maternal anemia, and a third was excluded because the fetus exhibited ventricular bigeminy on ultrasound examination prior to starting the study. One subject experienced uterine contractions and did not complete the study, bringing the total number of subjects who completed Phase II of the study to 19. There were no other trial terminations due to adverse events.

The mean age of the 19 subjects in Phase II was 30.0 (±0.87) years, their average gestation was 30.1(±0.28) weeks, and mean BMI was 26.6 kg/m^2^ (±1.4). Of the cases, 10 were primigravidas and 9 were multigravidas. There were 13 nurses, 6 homemakers, and 9 women doing administrative work.

### Effect on tidal volumes, breathing frequency, minute ventilation

**Fig. 4:**
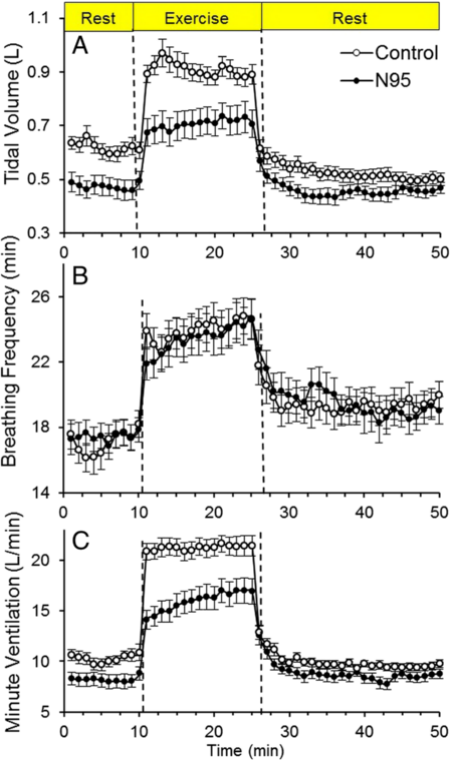
Ventilation functions in pregnancy during rest and exercise. Inspired and expired air volumes were measured with a pneumotachometer attached to mask outlet to obtain (**a**) tidal volume, (**b**) breathing frequency and (**c**) minute ventilation. *N* = 19 (±SEM)

During the pre-exercise rest period, breathing through N95-mask materials lowered TV by a mean of 0.15 L compared with controls (95 % CI: −0.23, −0.08; *p* < 0.001) (Fig. 4a). TV during both exercise cycles increased rapidly to reach a plateau which was about 50 % higher than that observed at rest, within a minute. However, compared with controls, exercising with N95-masks reduced mean TV by 0.21L (95 % CI: −0.32, −0.10; *p* < 0.001), a 23 % decrease (Fig. 4a, Table 1). Mean BF increased by 35 % during exercise for both control and N95 cycles compared to the rest period, but there was no difference in BF with and without wearing N95-masks (Fig. 4b, Table 1). Wearing N95-respirators lowered V_E_ by 25.8 %, a mean difference of 5.55L/min (95 % CI: −7.58, −3.51; *p* < 0.001) (Fig. 4c, Table 1). Significant differences in TV and V_E_ with N95 cycles persisted in the post-exercise rest period (Table 1). There was a tidal volume reduction of 0.08L (*p* = 0.02) and minute ventilation reduction of 1.1L/min (*p* = 0.031).

**Table 1:** Changes in respiratory parameters of pregnant subjects breathing through N95 masks compared to controls breathing ambient air

N95 Mask vs Control	Mean difference	SE (95 % CI)	*P* value
Pre exercise rest period			
VO_2_	−0.40	0.30 (−1.02, 0.23)	0.20
VCO_2_	−0.04	0.02 (−0.07, −0.003)	**0.035**
Tidal volume	−0.15	0.04 (−0.23, −0.08)	**< 0.001**
Breathing frequency	0.31	0.69 (−1.13, 1.75)	0.66
Minute ventilation	−2.23	0.66 (−3.60, −0.85)	**0.003**
FeO_2_	−0.52	0.97 (−0.79, −0.25)	**0.001**
FeCO_2_	0.25	0.07 (0.11, 0.40)	**0.002**
Fi O_2_	−0.02	0.07 (−0.16, 0.12)	0.76
Fi CO_2_	−0.01	0.01 (−0.03, 0.01)	0.47
Exercise period			
VO_2_	−1.30	0.47 (−2.3, −0.31)	**0.01**
VCO_2_	−0.10	0.25 (−0.15, −0.05)	**0.001**
Tidal volume	−0.21	0.05 (−0.32, −0.11)	**< 0.001**
Breathing frequency	−0.51	0.67 (−1.92, 0.89)	0.45
Minute ventilation	−5.55	0.97 (−7.58, −3.51)	**< 0.001**
FeO_2_	−0.54	0.08 (−0.70, −0.38)	**< 0.001**
FeCO_2_	0.30	0.06 (0.18, 0.42)	**< 0.001**
Fi O_2_	0.02	0.08 (−0.13, 0.17)	0.81
Fi CO_2_	0.004	0.01 (−0.23, 0.03)	0.75
Post exercise rest period			
VO_2_	−0.22	0.21 (−0.67, 0.23)	0.32
VCO_2_	−0.01	0.01 (−0.04, 0.01)	0.29
Tidal volume	−0.08	0.03 (−0.14; −0.01)	**0.02**
Breathing frequency	0.48	0.50 (−0.56, 1.53)	0.34
Minute ventilation	−1.10	0.47 (−2.10, −0.11)	**0.031**
FeO_2_	−0.30	0.09 (−0.49, −0.11)	**0.004**
FeCO_2_	0.19	0.05 (0.09, 0.29)	**0.001**
Fi O_2_	−0.03	0.09 (−0.2, 0.16)	0.77
Fi CO_2_	0.00	0.02 (−0.03, 0.03)	0.99

(bolded values: statistically significant)

### Effect on O_2_ and CO_2_ concentrations in inspired and expired air

**Fig. 5:**
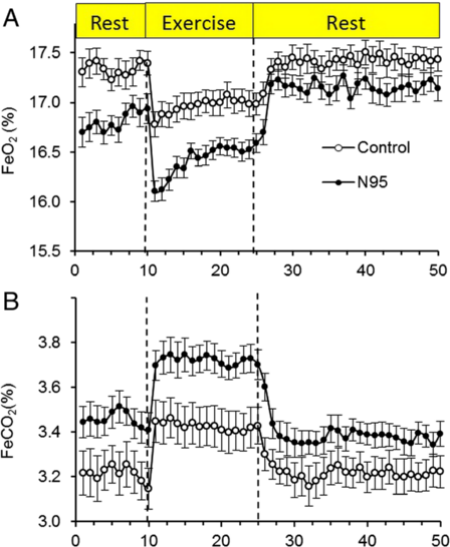
Oxygen and Carbon dioxide content of expired air in pregnant women at rest and following exercise. (**a**) Forced expired O_2_ (FeO_2_) and (**b**) forced expired CO_2_ concentrations (FeCO_2_) were measured with a metabolic cart. *N* = 19 (±SEM)

**Fig. 6:**
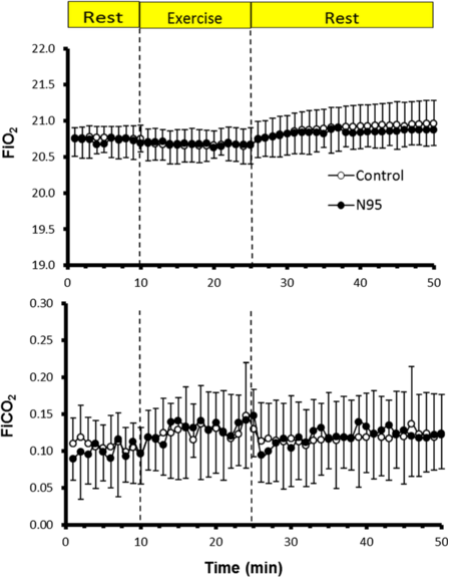
Oxygen and Carbon dioxide content of inspired air in pregnant women at rest and following exercise. (**a**) Forced inspired O_2_ (FiO_2_) and (**b**) forced inspired CO_2_ concentrations (FiCO_2_) were measured with a metabolic cart. *N* = 19 (±SEM)

Forced expired O_2_ concentration (FeO_2_) during the pre-exercise rest period decreased by 0.52 % (95 % CI: −0.79, −0.25; *p* = 0.001) with N95-mask use versus controls (Fig. 5a, Table 1). Wearing of N95-masks during exercise reduced FeO_2_ by 0.54 % compared with controls (95 % CI: −0.70, −0.38; *p* < 0.001). Reduction in FeO_2_ with the use of N95-masks persisted in the post-exercise rest period. Concomitantly, forced expired CO_2_ concentration (FeCO_2_) was significantly elevated with N95-mask use in the pre-exercise, exercise and post exercise periods compared with no mask usage (Fig. 5b, Table 1). During the exercise period, the wearing of N95-masks resulted in increase in FeCO_2_ of 0.30 % (95 % CI: 0.18, 0.42; *p* < 0.001) compared with controls. (Fig. 5b, Table 1). In contrast, no significant differences were observed in the inspired oxygen (FiO_2_) or carbon dioxide (FiCO_2_) concentrations of inspired air before, during or after exercise (Fig. 6, Table 1).

### Effect on pulmonary gas exchange

**Fig. 7:**
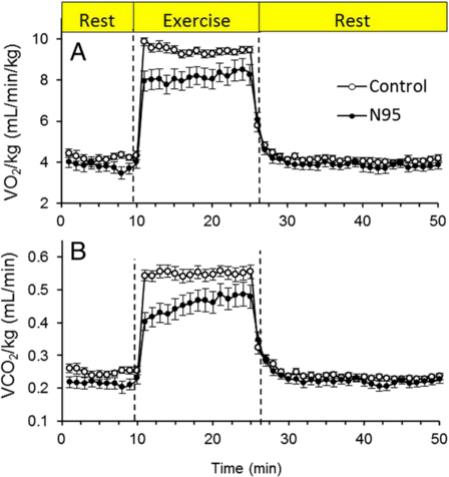
Pulmonary gas exchange in pregnant women at rest and following exercise. O_2_ and CO_2_ concentrations were measured with a metabolic cart to calculate volumes of oxygen (**a**) VO_2_, or carbon dioxide (**b**) VCO_2_ exchanged with each breath. *N* = 19 (±SEM)

When performing work on the treadmill equivalent to 3 MET, VO_2_ and VCO_2_ increased by about two-fold for all subjects compared to the rest periods. Strikingly, wearing of the N95-mask during exercise resulted in lowering VO_2_ by 13.8 %, a mean of 1.30 ml/min/kg (95 % CI: −2.30, −0.31; *p* = 0.013) (Fig. 7a, Table 1). Similarly, VCO_2_ was lowered by 17.7 %, a mean of 0.10 ml/min/kg (95 % CI: −0.15, −0.05; *p* = 0.001) (Fig. 7b, Table 1).

### Effect on maternal and fetal physiological parameters

For all subjects, overall maternal heart rate increased from 89 ± 1.8 to 107 ± 1.9 beats/min with exercise. There were no significant difference in heart rate between the N95-masks and control cycles. There were also no changes in basal fetal heart rates (mean heart rate of 133 beats per minute) or variability (15–16 beats per minute) in all the CTGs. There were no significant differences in lactate levels pre exercise (1.8 ± 0.2 mmol/L), post exercise breathing through ambient air (1.6 ± 0.2 mmol/L), and post exercise with the N95-mask (2.1 ± 0.4 mmol/L). There were no differences in finger-tip capillary oxygen saturation levels with the mask and without the mask; 98.3 ± 0.18 % and 98.4 ± 0.11 % respectively. The Borg scale indicated that exercise induced a borderline increase in perceived effort from being 9.1(±0.60) to 10.7(±0.8) after the N95 cycles. These parameters did not reach statistical significance (Table 2).

**Table 2:** Changes in maternal and fetal physiological parameters breathing through N95 masks compared to controls breathing ambient air

N95 Mask vs Control	Mean difference	SE (95 % CI)	*P* value
Pre exercise maternal heart rate	−2.0	1.3 (−4.7; 0.6)	0.11
Exercise maternal heart rate	−0.5	1.4 (−3.4; 2.4)	0.71
Post exercise maternal heart rate	−0.8	1.6 (−4.1; 2.5)	0.61
Baseline fetal heart rate	−0.5	0.6 −1.6; 0.6)	0.33
Capillary lactate	0.5	0.5 (−0.3; 1.4)	0.17
Finger-tip capillary oxygen saturation	0.1	0.02 (−0.06; 0.14	0.32
Borg scale	1.6	0.9 (−0.01; 3.3)	0.060

## Discussion

We found that in women in mid-pregnancy, breathing through the N95 respirator material when performing low intensity work significantly reduced VO_2_ (13.8 %) and VCO_2_ (17.7 %), which was due to a corresponding decrease in V_E_ (25.8 %) and TV (23 %), without a compensatory increase in BF. This decrease in air intake volume, together with unchanged concentrations of inspired O_2_ and CO_2_ imply a decrease in overall amount of O_2_ and CO_2_ inspired. Coupled with a 3.2 % decrease in FeO_2_ and an 8.9 % increase in FeCO_2_, these results suggest an increased consumption of O_2_ and production of CO_2_, leading to possible concerns regarding prolonged usage of N95-masks on respiratory functions in pregnant women performing physical work. The decrease in V_E_, TV, FeO_2_ and the increase in FeCO_2_ were also significant during the rest periods. These results suggest that breathing through the N95 mask material can limit the overall volume of amount of oxygen intake and also increase the rate of metabolism.

When performing work equivalent to routine bedside nursing with N95-masks, non-pregnant subjects have been previously reported to maintain their V_E_ compared with controls, with non-significant changes in the TV and BF [26]. Other studies in both non-pregnant and pregnant subjects have shown, on the contrary, an increase or decrease in BF but the TV and V_E_ were not measured in these trials [17, 27, 28]. In contrast, our pregnant subjects were unable to proportionately increase both TV and BF to maintain their V_E_ during rest and in response to exercise while breathing through N95-masks materials. A significant 26 % reduction in V_E,_ with a 23 % reduction in mean TV was noted during exercise, possibly due to diaphragmatic splinting. This decrease in V_E_ led to a corresponding decrease in VO_2_ (13.8 %) and VCO_2_ (17.7 %) (Fig. 7). The decrease in FeO_2_ and increase in FeCO_2_ are likely due to a stimulation of the respiratory drive resulting in greater efforts required to breathe through the N95 mask materials and a concomitant greater extraction of O_2_ for aerobic metabolism. These results also suggest that the N95 mask may impede gaseous exchange resulting in hypoventilation. Although the subjects appeared to adapt to the increased workload with no changes in the other maternal and fetal physiological parameters with no evidence of hypoxia found on finger-tip capillary oxygen saturation nor increased lactic acid production to suggest a shift toward anaerobic respiration, our results suggest that performing physical work with the N95-mask appears to stress the aerobic metabolism and increase the CO_2_ load within the circulation.

In non-pregnant subjects, it has been shown that use of N95-respirators can increase CO_2_ levels within the masks by 1.8–3 %, suggesting that the increase in expired CO_2_ concentration could also be due to the accumulation of expired CO_2_ trapped in the dead space of the N95 mask [29, 30]. Our results do not support such a view because FiCO_2_ did not increase even with the use of N95 materials and total CO_2_ intake was reduced due to the corresponding decrease in V_E_. These results further affirm that the increase in expired CO_2_ mainly arose from increased rate of aerobic metabolism. The higher circulating CO_2_ concentration observed in our study was in agreement with increase in transcutaneous CO_2_ observed in pregnant and non-pregnant women after a 20-minute exercise wearing a respirator as compared to not wearing the respirator [17]. It must be borne in mind that CO_2_ levels in the blood of pregnant women are usually lower due to physiological hyperventilation. An increase in forced expired CO_2_ which reflects a rise in blood CO_2_ levels hence contributes to impair elimination of fetal CO_2_ as arterial CO_2_ is normally reduced in pregnancy to allow for a steeper diffusion gradient of CO_2_ from fetal blood across to the mother.

Our study was limited in that we were unable to evaluate the effects of N95-mask usage at higher work intensities and over longer durations because of ethical concerns. To ascertain safety, parameters such as maternal and fetal heart rate changes, lactate and finger-tip capillary oxygen saturation levels were monitored to ensure that no significant hypoxemia was induced in our subjects and their fetuses.

Wearing the Hans Rudolph mask with aperture occluded by the mask material allows accurate measurement of respiratory parameters despite its use not being exactly the same as the standard use of the N95-mask. However this is the closest way of obtaining accurate physiologic data to best characterize the impact of the materials used in the respirators. The other limitation of our study was the narrow window of gestation studied (between 27 to 32 weeks). These group of women were deemed to be representative of pregnant women as they would have undergone marked respiratory adaptations to pregnancy, especially diaphragmatic splinting from the enlarging uterus. However, it is postulated that, in situations of exercising at a higher intensity, prolonged N95-mask usage or at more advanced gestations, a greater degree of oxygen deficit due to a corresponding decrease in V_E_ can have more marked effects on the respiratory function of pregnant women. We also focused exclusively on pregnant women without using non-pregnant controls to better define the risks in this group which are the most controversial.

Our study demonstrates, for the first time, that pregnant women in mid-pregnancy are unable to maintain their minute ventilation while breathing through N95-mask materials. There is also a decrease oxygen uptake and increase carbon dioxide production as a result of the increased workload on breathing imposed by mask use, both at rest and low work intensity. This supports the view of some that pregnant healthcare workers should probably refrain from prolonged N95-mask use towards the third trimester. The complications to the women and her fetus that can result from a prolonged decrease in V_E_ and increased work of breathing, are unknown.

## Conclusions

While there is a substantial negative change in TV, V_E_ and the VO_2_ and VCO_2_ exchanged, there was no impact of breathing through N95 mask materials, to finger-tip oxygen saturation, maternal or fetal heart rate and no drive to increase BF in pregnancy compared to breathing ambient air at the level of exercise in our study. This study shows important descriptive findings of changes to respiratory physiology with mask use, which do not appear to have sufficient significant clinical impact based on the parameters monitored, that had been deliberately kept within the normal ranges to ensure safety of the subjects. Although harm was not demonstrated in the context of this experimental protocol, the significant changes to respiratory physiology caused by breathing through N95 mask materials raise the concern regarding prolonged use of N95-masks by pregnant healthcare workers. Our results suggest that pregnant women may experience more fatigue and require more rest breaks from mask use. Scheduled work breaks should be considered for pregnant healthcare workers working in high risk areas which require prolonged use of N95 respirators. In face of the imminent threat of pandemic airborne respiratory diseases it should be emphasized that the benefit of using N95 mask to prevent serious emerging infectious diseases should be weighed against possible respiratory consequences associated with N95 mask usage. Use of alternative protective methods such as surgical masks with lesser airway resistance, should be considered in appropriate settings [31–33]. These have been shown to be equally effective for the prevention of droplet infections such as influenza [31], although they are insufficient for protection against airborne pathogens. Innovative interventions to improve the design of FFR, are urgently needed in the face of the imminent threat of pandemics of acute airborne respiratory infections. The key is to ensure healthcare worker protection from infectious agents without jeopardizing the wellbeing of the pregnant healthcare workers and their fetuses.
