# Erratum to: Respiratory consequences of N95-type Mask usage in pregnant healthcare workers—a controlled clinical study

**DOI:** 10.1186/s13756-016-0125-4

**Published:** 2016-07-04

**Authors:** Pearl Shuang Ye Tong, Anita Sugam Kale, Kailyn Ng, Amelia Peiwen Loke, Mahesh Arjandas Choolani, Chin Leong Lim, Yiong Huak Chan, Yap Seng Chong, Paul Anantharajah Tambyah, Eu-Leong Yong

**Affiliations:** Department of Obstetrics and Gynecology, National University Hospital, 11 Mandalay Road, Singapore, 308232 Singapore; Lee Kong Chian School of Medicine, Nanyang Technological University, 11 Mandalay Road, Singapore, 308232 Singapore; Biostatistics Unit, National University of Singapore, Singapore, Republic of Singapore; Medicine, National University of Singapore, 1E Kent Ridge Road, Level 12, Singapore, 119228 Singapore

## Erratum

The original article [1] listed the clinicaltrials.gov identifier in the Trial Registration sub-section as NCT00265926; this identifier should instead have read ‘NCT02265926’.
